# Spectrophotometric analysis evaluating apical microleakage in retrograde filling using GIC, MTA and biodentine: an in-vitro study

**DOI:** 10.1186/s12903-020-1025-9

**Published:** 2020-02-03

**Authors:** Manisha Nepal, Snigdha Shubham, Rupam Tripathi, Jwolan Khadka, Deepa Kunwar, Vanita Gautam, Narayan Gautam

**Affiliations:** 1 Department of Conservative Dentistry and Endodontics, Universal College of Medical Sciences, Bhairahawa, Nepal; 2 Department of Conservative Dentistry and Endodontics, KIST Medical College, Lalitpur, Nepal; 3 Department of Conservative Dentistry and Endodontics, Gandaki Medical College, Pokhara, Nepal; 4 Department of Biochemistry, Universal College of Medical Sciences, Bhairahawa, Nepal

**Keywords:** Apical microleakage, Apicoectomy, Biodentine, GIC, MTA, Root-end filling materials

## Abstract

**Background:**

The present study compares the apical microleakage of three different root-end filling materials in which the retrograde cavity is prepared by two different burs.

**Methods:**

Eighty extracted single rooted maxillary and mandibular premolars were taken. Root canal treatment was completed. Apical 3 mm of all the teeth were resected with diamond disk. The tooth were divided into four groups with two subgroups for each group containing 10 tooth (*N* = 10) as: Group IA (Negative Control and IB (Positive Control); Group IIA and IIB: Prepared with round carbide bur and round diamond bur respectively, filled with GIC; Group IIIA and IIIB: Prepared with round carbide bur and round diamond bur respectively, filled with MTA; Group IVA and IVB: Prepared with round carbide bur and round diamond bur, filled with Biodentine. After applying two coats of nail varnish leaving apical 3 mm (except for negative control group) all teeth were immersed in 2% methylene blue for 3 days and again in 65% nitric acid for next 3 days for extraction of dye. The obtained solution was then transferred to eppendorf tube and centrifuged in microcentrifuges at 14,000 revolution per minutes (RPM) for 5 min. Optical density or absorbance of the supernatant solution was measured with UV spectrophotometer at 550 nm.

**Results:**

The absorbance of the supernatant solution after dye extraction is decreasing in the order of positive control> GIC > MTA > Biodentine> negative control group. The significant difference was observed between GIC and MTA (*p* = 0.0001) and GIC and Biodentine (*p* = 0.0001) with two different burs but statistically non-significant difference was observed between MTA and Biodentine with Carbide bur (*p* = 0.127) and Diamond bur (*p* = 0.496) respectively.

**Conclusions:**

Within the limitations of the present study, it can be concluded that Biodentine and MTA showed less microleakage as compared to GIC. There is no significant difference between mean microleakage of MTA and Biodentine. However, the mean OD of the Biodentine was least of all evaluated materials. Preparation of the root-end using round carbide bur as well as round diamond burs showed comparable microleakage for all three filling materials.

## Background

Conventional Root Canal Treatment (RCT) is the highly predictable treatment option with the aim of elimination and future exclusion of all the microorganisms from the root canal system [1]. The most important factor to achieve this is complete removal of infected canal contents followed by obliteration of the root canal system and development of fluid tight seal. Nonsurgical endodontic treatment is a predictable and reliable treatment with high success rates ranging from 86 to 98% [2]. In some cases, despite meticulous canal cleaning, shaping, disinfection, and obturation, endodontic treatment might still fail. This unsuccessful result may related to bacterial persistence in the apical canal in areas unaffected by treatment procedures [3]. Procedural errors during instrumentation like ledges, perforations and instrument breakage, canal calcifications and anatomic anomalies can negatively affect the efficient performance of cleaning and shaping of the root canal system and lead to treatment failure [4, 5].

Nonsurgical retreatment is the preferred treatment option if conventional endodontic treatment is unsuccessful. According to Bergenholtz et al. [6] this treatment usually results in successful outcomes. However, ideal goals may be difficult to achieve with a retreatment approach because of the complexity of root canal systems, inadequate instrumentation and presence of physical barriers viz. a viz. anatomical, post and core restoration, separated instruments, etc. Hence, in such cases surgical endodontic therapy becomes the first alternative to save the involved tooth.

The teeth committed with persistent periapical lesion in which root canal retreatment had failed or is not feasible is salvaged by apicoectomy which is a well-established surgical procedure. Saving the naturals is our prime concern so this procedure is an alternative to avoid extractions. It is an important conservative treatment and an extension of endodontic therapy whose purpose is to preserve the tooth [7].

Apicoectomy procedure includes exposure of the involved root apex, curettage of the lesion,root-end resection, root-end cavity preparation and root-end filling. The concern of apicoectomy is not just only the removal of the diseased periapical tissue and apex of root, but also, resealing the root canal system with a suitable root-end filling material.

Gutta Percha, Amalgam, Cavit, Intermediate Restorative Material, Super EBA, Diaket, GlassIonomer Cements, Composite Resins, Carboxylate Cements, Zinc Phosphate Cements, Zinc Oxide Eugenol Cements etc. had been used traditionally as a root end filling material [8]. In 1993, MTA was developed as a new root-end filling material at Loma Linda University, California, USA. It is regarded as an ideal root-end filling material and has become the gold standard against which the newer materials are compared [9]. Other newer bioceramic materials like BioAggregate, Biodentine, Endosequence root repair material (ERRM), iRoot BP Plus, etc. have been marketed recently.

Apical microleakage is the leakage along the interface between the filling material and the canal wall [10]. Apical leakage continues to be a topic of interest because in spite of advances in endodontics, clinical failure still occurs. The purpose of root-end filling material is to entomb surviving microbes in the root canal space so that they cannot multiply and/or communicate with the periradicular tissues, also to prevent influx of periapical fluids which nourish surviving microbes in the root canal [11]. Inappropriate marginal sealing of retrocavity may allow percolation of microorganisms and their products between the root canal system and periradicular tissues, thus leading to treatment failure.

The quality of apical seal achieved by root-end filling materials has been assessed by various means like the degree of dye penetration, dye extraction, radioisotope penetration, bacterial penetration, electro-chemical means and fluid filtration techniques. Dye extraction method employs the immersion of samples in dye followed by acid that liberates all of the dye from within the interface, and the optical density of the solution is recorded by the use of a spectrophotometer. Thus, it is possible to quantitatively measure how much dye penetrates through the margins of restoration [12].

Since the adequacy of the “apical seal” is considered paramount to the success of apicectomies, the sealing ability of a new material must be evaluated [1]. Therefore, this study is aimed to evaluate the apical sealing ability of GIC, MTA and Biodentine as a root-end filling material by preparing retrograde cavity with two different burs: round carbide bur and round diamond bur by utilizing dye extraction method for leakage assessment.

## Methods

The study was conducted in Department of Conservative dentistry and Endodontics from March 2017 to August 2017 with due approval of Institutional Review Committee (UCMS/IRC/092) of Universal College of Medical Sciences, Bhairahawa, Nepal. The consent of the participant for the use of extracted tooth is not necessary according to the Institutional Review Committee which comes under National regulation (Nepal). Eighty intact human maxillary and mandibular premolars with single root and canal extracted for orthodontic purpose from the patients of the similar age group, caries free, similar dimensions, without signs of fractures/cracks and with similar tooth length were selected as a sample. Teeth were immersed in 5% sodium hypochlorite solution for 5 min and ultrasonic scaler was used to remove soft tissues, calculus and any external debris from the teeth, then stored in a container containing 10% buffered formalin solution until further use.

Access opening was done with Endo Access burs (Dentsply) and working length determined with #15 K file (Diadent), confirmed by Radiovisiograph (RVG). Chemomechanical preparation was done using hand K files (#40, 2% taper) with step-back technique, irrigation done with 5% NaOCl (Pyrax, India), 17% EDTA (Ammdent) and normal saline. Canal was dried with paper points and obturation done with gutta percha (Diadent) and zinc oxide eugenol sealer using lateral condensation technique with finger spreader. A final radiograph was taken using RVG that demonstrated adequately the obturation of each canal. Access cavity was restored with Cavit. Samples were stored in 100% relative humidity for 24 h. Apical 3 mm of all the teeth were resected at 90° angle axis to the long axis of the root with diamond disk mounted in straight handpiece of micromotor. Samples were divided into four groups with two subgroups of 10 sample each (*N* = 10) as:

- Group I: Control Group.
- Group IA (Negative Control): without root-end preparation and without root-end filling; all surface covered with nail varnish.
- Group IB (Positive Control): without root- end preparation and without root-end filling.
- Group II: Root-end filling with GIC, where retrograde cavity is prepared with.
- Group IIA: round carbide bur.
- Group IIB: round diamond bur.
- Group III: Root-end filling with MTA, where retrograde cavity is prepared with
  Group IIIA: round carbide bur.
- Group IIIB: round diamond bur.
- Group IV: Root-end filling with Biodentine where retrograde cavity is prepared with.
- Group IVA: round carbide bur.
- Group IVB: round diamond bur.

Retrograde preparations were made in all the teeth uniformly to a depth of 3 mm using two different burs: round carbide bur and round diamond bur (SS White). Root-end cavity was irrigated with normal saline, dried with absorbent paper points and restored with GIC (Shofu, Japan), MTA (MTA Angelus^R^, Brazil) and Biodentine™ (Septodont, France) in the respective groups. Samples were coated with two coats of nail varnish leaving apical 3 mm (except Group IA where all surface was covered with nail varnish). After the varnish was dried, samples were immersed in 5 ml of 2% methylene blue in 80 different air tight container and stored in incubator at 37 °C for 72 h. Samples were washed under running tap water to remove the traces of the dye, dried and nail varnish removed with scalpel. Each sample were immersed and stored in test tube containing 1 ml of freshly prepared 65% HNO_3_ for 72 h. The obtained solution was transferred to effendorf tubes and centrifuged at 14,000 RPM for 5 min to separate GP debris from the extracted dye. Dye concentration in the supernatant solution was analyzed using an UV spectrophotometer at 550 nm taking concentrated nitric acid as a blank.

Statistical analysis was performed using Statistical Package for the Social Sciences (SPSS) version 20.00 (SPSS Inc., Chicago IL) to compare the mean apical microleakage of the groups and determine the significance of differences between different groups. Quantitative statistical analysis was done for the parameters. Mean and standard deviation was calculated for all scores obtained from 8 different study subgroups of four groups. One way ANOVA followed by post hoc Tukey HSD was used to analyse to compare mean ± SD. *p* < 0.05 was considered to be statistically significant.

## Result

**Fig. 1:**
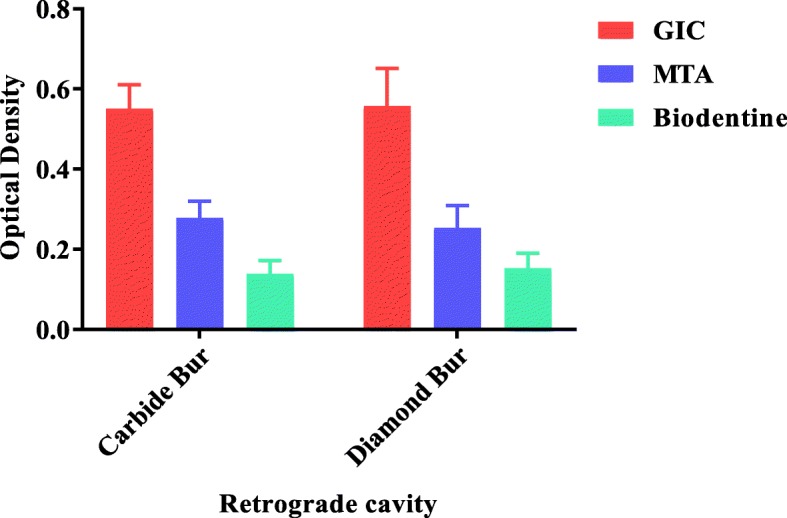
Comparison of (Mean ± S.D) Optical Density of three different root-end filling materials by two different burs. Graph 1 depicts mean ± S.D. OD for samples filled with GIC, MTA and Biodentine in which retrograde cavity was prepared with two different round burs: Carbide versus Diamond respectively. The significant difference was observed in between GIC and MTA (*p* = 0.0001) as well as GIC and Biodentine (p = 0.0001) with two different burs but statistically non-significant difference was observed between MTA and Biodentine with Carbide bur (*p* = 0.127) and Diamond bur (*p* = 0.496) respectively. While comparing Mean ± S.D. for two different round burs for retrograde cavity preparations (Carbide versus Diamond) of three different materials, statistically non-significant difference was observed between intra-groups GIC-Carbide versus GIC-Diamond (*p* = 1.000), MTA-Carbide versus MTA-Diamond (*p* = 1.000) and Biodentine-Carbide versus Biodentine-Diamond (*p* = 1.000).

Quantitative statistical analysis was done for the parameters. Mean and standard deviation was calculated for all scores obtained from 8 different study subgroups of four groups viz. a viz. (I) Control Group (Negative, Positive), (II) GIC Group (Carbide, Diamond), (III) MTA (Carbide, Diamond), (IV) Biodentine (Carbide, Diamond). Comparison of microleakage among 8 subgroups based on Optical Density (OD) showed least microleakage in Biodentine group prepared by carbide bur as depicted in Table 1 and Fig. 1.

**Table 1:** Comparison of microleakage among 8 subgroups based on Optical Density (OD)

Groups	Mean	S.D	N
Group IA	0.130	0.029	10
Group IB	0.948	0.287	10
Group IIA	0.550	0.060	10
Group IIB	0.557	0.095	10
Group IIIA	0.278	0.042	10
Group IIIB	0.254	0.055	10
Group IVA	0.139	0.033	10
Group IVB	0.153	0.038	10
Total	0.376	0.292	80

Shows the mean ± S.D. OD for different subgroups to assess microleakage

One way ANOVA followed by post hoc Tukey HSD was used to analyse to compare mean ± SD between the groups with positive and negative controls which also showed Biodentine to have least microleakage in comparison to negative control as shown by Table 2.

**Table 2:** Comparative analysis of microleakage with negative control and positive control

Groups	Mean (OD) ± S.D	Negative Control Mean (OD) ± S.D	*P*-value	Positive Control Mean (OD) ± S.D	*P*-value
Group IIA	0.550 ± 0.060		0.0001		0.0001
Group IIB	0.557 ± 0.095		0.0001		0.0001
Group IIIA	0.278 ± 0.042		0.084		0.0001
Group IIIB	0.254 ± 0.055	0.130 ± 0.029	0.234	0.948 ± 0.287	0.0001
Group IVA	0.139 ± 0.033		1.000		0.0001
Group IVB	0.153 ± 0.036		1.000		0.0001

Illustrates the comparison of mean ± S.D. of OD for samples filled with GIC, MTA and Biodentine in which retrograde cavity was prepared by two different round burs: Carbide versus Diamond with that of negative and positive control group. The significant difference was observed between GIC and negative control (*p* = 0.0001) and GIC and positive control (*p* = 0.0001). The non-significant difference was observed between MTA and negative control (*p* = 0.084/Carbide, *p* = 0.234/Diamond) and the significant difference between MTA and positive control (*p* = 0.0001). Moreover, the non-significant difference was observed between Biodentine and negative control with both burs (*p* = 1.000) and significant difference observed between Biodentine and positive control (*p* = 0.0001) respectively.

## Discussion

Endodontic surgery may become the last resort for salvaging the affected tooth, if conventional endodontic treatment fails and retreatment is neither indicated nor feasible. Removal of the infected root-end and sealing any remaining bacteria in the root canal system from the periradicular tissues is the goal of surgical intervention [13]. The surgical procedure includes three critical steps to eliminate persistent endodontic pathogens: 1) surgical debridement of pathological periradicular tissue, 2) root-end resection (apicoectomy), and 3) retrograde root canal obturation (root-end filling).

Kim S and Kratchman S suggested removing at least 3 mm of the root-end which reduces 98% of the apical ramifications and 93% of the lateral canals [14]. They also proposed that root-end amputation of less than 3 mm does not remove all of the lateral canals and apical ramifications which poses a risk of reinfection and eventual failure. Apart from amount, the plane of sectioning is to be considered equally during root resection. Ideally, the short bevel (0°) that is as perpendicular to the long axis of the tooth as possible conserves the root length and exposes less dentinal tubules thereby opening less tubules to be exposed to the environment, which allows less microleakage over a period of time [15].

Taschieri S et al. investigated the quality of root-end filling in cases of periapical lesions persisting after endodontic surgery. Failure of apicoectomy was because of an imperfect seal at the interface between the root-end filling and the cavity margin. The presence of such a gap would favour a continuous bacterial leakage from the infected root canal system to the periapical tissue thereby sustaining inflammation [16]. According to Cohen, the ideal root-end filling material should seal the contents of the root canal system within the canal, prevent egress of any bacteria, bacterial by-products, or toxic material into the surrounding periradicular tissues [17]. Various studies have emphasized the importance of root-end fillings in outcomes of apicoectomies by reporting that teeth with root-end fillings showed favorable results compared with those without root-end fillings [18–20]. Therefore, sealing the root apex with a proper root-end filling material is crucial.

Various experiments can be carried out to evaluate the microleakage like dye extraction, dye penetration, radioisotope, bacterial penetration, fluid filtration etc. The dye penetration method used for measuring sealing ability is the popular and most widely used but this technique suffers from severe limitations. This technique relies on randomly cutting the root into two pieces, without knowing if the section goes through the deepest dye penetration so it under evaluates the dye penetration and gives randomly chosen results [12]. Apart from that, the measurement of leakage is qualitative too. Whereas, in dye extraction method, all the dye that leaked through the apex is recovered by dissolving in acid which avoids the limitations of sectioning the root and it also quantitatively measures the optical density of the solution by the use of a spectrophotometer thus provides reliable results in microleakage studies [21]. However, sample storage in 10% formalin may introduce a significant source of experimental variability influencing leakage results as compared to freshly extracted teeth [22].

In the present study, the mean ± S.D. OD for GIC-Carbide as compared to MTA-Carbide and Biodentine-Carbide was found to be statistically significant difference (*p* = 0.0001). Similarly, the mean ± S.D. OD for GIC-Diamond as compared to MTA-Diamond and Biodentine-Diamond was also statistically significant difference (p = 0.0001). This result shows that the microleakage occurs more in one material as compared to others. However, while comparing GIC with negative control, it shows the significant difference in the microleakage butcomparing MTA and Biodentine with negative control it shows statistically non-significant difference representing that MTA and Biodentine have better apical seal as compared to GIC.

Moreover, in the present study the mean ± S.D. OD for MTA-Carbide (0.278 ± 0.042) and Biodentine-Carbide (0.139 ± 0.033) was statistically non-significant difference (*p* = 0.127). Similarly, the mean ± S.D. OD for MTA-Diamond (0.254 ± 0.055) and Biodentine-Diamond (0.153 ± 0.038) was also statistically non-significant difference (*p* = 0.496) showing the comparability of both materials in providing apical seal. In the same way, mean ± S.D. OD of MTA-Carbide and MTA-Diamond with were statistically non-significant difference as compared to negative control (*p* = 0.084 and *p* = 0.234) respectively. Likewise, mean ± S.D. OD of Biodentine-Carbide and Biodentine-Diamond were also statistically non-significant difference as compared to negative control (*p* = 1.000). Hence, MTA and Biodentine had similar apical sealing ability as observed by its optical density near to negative control which was statistically non-significant. Apart from that, the mean ± S.D. OD of Biodentine was lower than that of MTA representing Biodentine is the best material for preventing apical microleakage.

Mineral Trioxide Aggregate has been proven to show less microleakage compared to other materials [5]. However, in this study the least microleakage was exhibited by Biodentine although the difference was not statistically significant. The result of our study is in concurrence with the study conducted by Khandelwal A et al. [23], Radeva E et al. [24], Naik MM et al. [25], Kokate SR et al. [8] Comparing the sealing ability of MTA and Biodentine as root-end filling material Khandelwal A et al. [23] concluded that Biodentine can be used as a replacement for MTA. The study by Radeva E et al. [24] concluded that Biodentine can be more effective as apical sealing material compared to MTA. Naik MM^25^ concluded that the apical seal obtained with Biodentine was superior to that obtained with MTA.

Similarly, Kokate SR et al. [8] compared the microleakage of MTA, GIC & Biodentine using dye penetration method under stereomicroscope. The results of their study showed that there was significantly less leakage in Biodentine when compared to MTA & GIC. This result is in agreement with the present study. However, the study conducted by Mandava P et al. [26] evaluated the apical microleakage of root-end cavities filled with MTA, Biodentine and LC GIC using two different cavity preparation techniques that is conventional bur preparation and ultrasonic tip preparation. The result of their study showed significantly less microleakage of MTA compared to Biodentine and LC GIC, which is in contrast to our study.

In the current study, two different burs were used for preparing retrograde cavity. The rationale behind using two different burs is to determine the effect of smear layer on microleakage. Surgical smear layer in endodontics is defined as a smear layer, which contains microorganisms and necrotic pulpal tissues which is formed on the dentinal surfaces, cut by the instruments during apicoectomy and retrograde cavity preparation [25]. Citric acid, EDTA, 35% orthophosphoric acid and BioPure MTAD™ (mixture of tetracycline isomer, acid and detergent) have been recommended for the removal of surgical smear layer but in our study no attempt was done to remove it as the retrograde cavity was just irrigated with normal saline. The thickness of the smear layer is also affected by type of the bur used. Several studies have demonstrated that carbide bur produces thinner smear layer compared to that of diamond bur [27–29]. However, the current study showed the mean ± S.D. OD of GIC-Carbide and GIC-Diamond, MTA-Carbide and MTA-Diamond and Biodentine-Carbide and Biodentine-Diamond respectively with statistically non-significant difference (*p* = 1.000). Hence, in this study, the comparable nature of two different retrograde preparations were observed and can be used according to ease.

As in vitro evaluation does not always reveal their in vivo performance, hence clinical testing are still required for higher impact of result.

## Conclusion

Biodentine and MTA showed less microleakage as compared to GIC. There is no significant difference between mean microleakage of MTA and Biodentine. However, the mean OD of the biodentine was least of all evaluated materials. Root end preparation either by round carbide bur or round diamond bur didn’t show any variation in microleakage.

## Data Availability

The datasets used and analyzed during the study are available from the corresponding author on reasonable request.

## Abbreviations

ANOVA: Analysis of Variance
EDTA: Ethylene Diamine Tetraacetic Acid
GIC: Glass Ionomer Cement
GP: Gutta Percha
MTA: Mineral Trioxide Aggregate
OD: Optical Density
RCT: Root Canal Treatment
RPM: Revolutions per minute
SD: Standard Deviation
UV: Ultraviolet
